# Short-course PET based simultaneous integrated boost for locally advanced cervical cancer

**DOI:** 10.1186/s13014-016-0612-z

**Published:** 2016-03-12

**Authors:** Marius Røthe Arnesen, Bernt Louni Rekstad, Caroline Stokke, Kjersti Bruheim, Ayca Muftuler Løndalen, Taran Paulsen Hellebust, Eirik Malinen

**Affiliations:** Department of Medical Physics, The Norwegian Radium Hospital, Oslo University Hospital, PO Box 4953, Nydalen, N-0424 Oslo, Norway; Department of Physics, University of Oslo, Oslo, Norway; The Intervention Centre, Oslo University Hospital, Oslo, Norway; Faculty of Health Sciences, Oslo and Akershus University College of Applied Sciences, Oslo, Norway; Department of Oncology, Oslo University Hospital, Oslo, Norway; Department of Radiology and Nuclear Medicine, Oslo University Hospital, Oslo, Norway

**Keywords:** Cervical cancer, Radiotherapy, Dose painting, PET, SIB, Dose escalation, Brachytherapy

## Abstract

**Background:**

Patients with large, locally advanced cervical cancers (LACC) are challenging to treat. The purpose of this work is to use 18F-FDG PET as planning basis for a short-course simultaneous integrated boost (SIB) in external beam radiotherapy of LACC in order to increase tumour shrinkage and likelihood of local control.

**Methods:**

Ten previously treated patients with LACC were included, all with pre-treatment FDG PET/CT images available. The FDG avid tumour volume, MTV_50_, was dose escalated in silico by intensity modulated radiotherapy from the standard 1.8 Gy to 2.8 Gy per fraction for the 10 first fractions; a short-course SIB. For the 18 remaining external fractions, standard pelvic treatment followed to total PTV and MTV_50_ doses of 50.4 Gy and 60.4 Gy, respectively. Photon and proton treatment were considered using volumetric modulated arc treatment (VMAT) and intensity-modulated proton therapy (IMPT), respectively. All treatment plans were generated using the Eclipse Treatment Planning System (TPS). The impact of tumour shrinkage on doses to organs at risk (OARs) was simulated in the TPS for the SIB plans.

**Results:**

Dose escalation could be implemented using both VMAT and IMPT, with a D_98_ ≥ 95 % for MTV_50_ being achieved in all cases. The sum of the 10 fraction short-course SIB and subsequent 18 standard fractions was compared to the standard non-SIB approach by dose volume histogram (DVH) analysis. Only marginal increase of dose to OARs was found for both modalities and a small further increase estimated from tumour shrinkage. Most DVH parameters showed a mean difference below 2 %. IMPT had, compared to VMAT, reduced OAR doses in the low to intermediate dose range, but showed no additional advantage in dose escalation.

**Conclusions:**

Planning of dose escalation based on a FDG avid boost volume was here demonstrated feasible. The concept may allow time for enhanced tumour shrinkage before brachytherapy. Thus, this strategy may prove clinically valuable, in particular for patients with large tumours.

## Background

Cervical cancer is one of the most common cancers among women worldwide [1]. Standard treatment for patients with locally advanced disease is external beam radiotherapy (EBRT) with concomitant chemotherapy followed by brachytherapy (BT). Good clinical outcome is achieved, but there are still about one in ten patients who suffer from local failure and typically two out of ten experience moderate to severe late side effects [2–4]. For BT, magnetic resonance (MR) based image guided adaptive brachytherapy (IGABT) is recommended as the best practice [5, 6]. Patients with large tumours at time of brachytherapy do however remain challenging to treat, particularly without combining intracavitary and interstitial implants and with limited imaging capability. The usage of interstitial implants is invasive and many centres worldwide do not offer this technique. Furthermore, there are many centres that still rely on 2D imaging for BT planning [7, 8]. It is therefore of interest to explore new EBRT strategies that may facilitate for improved brachytherapy by reducing the complexity and thus potentially improve the outcome for cervical cancer patients that are challenging to treat today.

18F-fluorodeoxyglucose (FDG) is the most widely used tracer for positron emission tomography (PET), especially in oncological imaging. FDG PET depicts glucose metabolism, which is increased in most malignant tumours [9]. FDG PET has proven valuable in pre-treatment assessment of cervical cancers, as both metabolic volume [10] and maximum standardised uptake value (SUV_max_) [11] are predictive of treatment response. Furthermore, in evaluating treatment response a sustained high FDG uptake during and after treatment may indicate continued presence of viable tumour cells and a poor prognosis [12, 13].

Today, PET/CT images of patients with cervical cancer are mainly utilized to detect metastatic disease [14] often found in pelvic and para-aortic lymph nodes with potential major impact on treatment strategies. Still, one may consider further exploiting information from the PET images about the primary tumour to guide radiotherapy for these patients. In the current work we propose to escalate the dose to a PET based metabolic target volume during the two first weeks of external beam therapy. In this short-course approach, a simultaneous integrated boost (SIB) technique using intensity modulation is proposed for dose escalation. The treatment concept is investigated for patients with locally advanced cervical cancer exploring both photon and proton intensity modulated radiotherapy. This novel concept for external beam dose escalation early in the treatment course may improve the subsequent brachytherapy by increasing tumour shrinkage. The treatment concept may have potential to improve outcome for patients with locally advanced cervical cancer.

## Methods

### Patient population

Ten patients with locally advanced cervical cancer were retrospectively selected for this study, all previously treated with curative intent according to standard clinical practice at our institution. Patients with intermediate to large primary tumours were selected with PET defined volumes ranging from 34 cm^3^ to 128 cm^3^. The cohort had a median age of 46 years ranging from 37 to 71 years with FIGO stages ranging from 2b to 3b. This study was approved by the regional research ethics committee (Regional Committees for Medical and Health Research Ethics, South East Norway) and informed consent was obtained from all patients.

### Standard treatment and imaging

Standard treatment consists of 50.4 Gy to the planning target volume (PTV) with 28 external beam fractions of 1.8 Gy, given 5 times a week. After around three weeks, additional image guided high dose rate (HDR) BT to the primary tumour is delivered in four fractions using intracavitary implants alone, or in combination with interstitial implants. Planning aims during brachytherapy, including EBRT dose, are a D_90_ (minimum dose to 90 % of the volume) of 85 Gy for the high risk CTV [5] (HR-CTV) and a D_98_ of 95 Gy for the gross target volume (GTV), in 2 Gy equivalent doses (EQD2) assuming α/β = 10. For organ at risks (OARs) overall D_2cm_^3^ constraints (minimum dose to the 2 cm^3^ volume receiving highest dose) are 70 Gy for rectum, sigmoid and small bowel and 80 Gy for the bladder (EQD2, assuming α/β = 3). The patients also receive concomitant chemotherapy with cisplatin (40 mg per m^2^) once a week to a total of 4–6 cycles depending on tolerance.

A treatment planning FDG PET/CT scan (Biograph, Siemens, Erlangen, Germany) over the pelvis and abdomen had been performed as part of clinical practice for all patients included in this study. The FDG PET scans are acquired 60–70 min after injection of 350 MBq (±20 %) activity. The OSEM reconstruction algorithm (8 iterations and 4 subsets) are used together with a 5 mm Gaussian smoothing. The resulting PET images have slice thickness and pixel size of 2.0 mm and 2.7 mm, respectively, while the CT images have 2.0 mm and a 1.0 mm, respectively. Medical images are imported to an Oncentra Treatment Planning System (Version 4.3, Nucletron, an Elekta company, Veenendaal, The Netherlands) for treatment planning. Target volumes and organs at risk are delineated by radiation oncologists and the primary tumour on PET images by nuclear medicine specialists. MR images are also routinely used in the definition of the primary tumor and pathological lymph nodes for these patients, but were not considered in this PET based study.

### Study concept

**Fig. 1:**
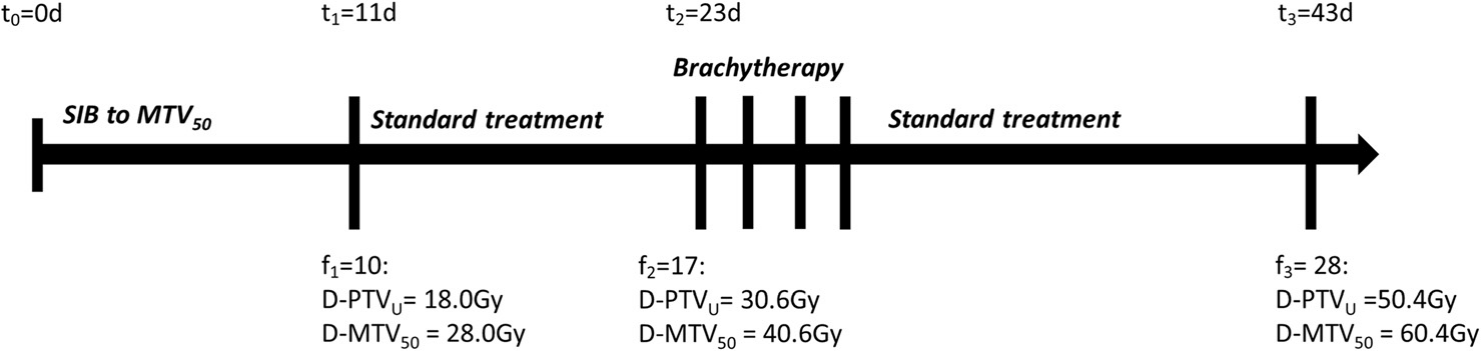
Timeline illustrating doses to MTV_50_ and PTV_union_ during fractionated treatment following the proposed treatment concept. In this example with a schedule of 5 fractions per week, the short course SIB is completed after 11 days, 12 days before onset of brachyterapy

To explore the potential of PET based EBRT dose escalation, a new treatment concept is suggested. A metabolic target volume, MTV_50_ within the primary tumour is defined by auto-segmentation using 50 % of SUV_max_ as a threshold. MTV_50_ is to be dose escalated by intensity modulated radiotherapy from the standard mean dose of 1.8 Gy to 2.8 Gy per fraction for the 10 first fractions; a short-course SIB. For the remaining 18 EBRT fractions, standard treatment to the pelvic area follows (Fig. 1). By the suggested concept, the dose to MTV_50_ is escalated from an EQD2 of 49.6 Gy to 61.7 Gy during EBRT. Consequently, assuming that MTV_50_ is fully covered by the GTV and planning aims met during BT, a total EQD2 D_98_ of 107.1 Gy may be achieved for MTV_50_. The feasibility of the short-course approach was tested in silico using both photon and proton techniques for intensity modulation.

Treatment planning for both photons and protons was performed using the Eclipse Treatment Planning System (v.11, Varian Medical Systems, Palo Alto, CA), after importing FDG PET/CT scans and RT structures. The central clinical target volume (CTV_central_) included a 5 mm isotropic expansion of the primary tumour (GTV_tumour_, defined from PET images), the uterus, both parametria and proximally 3 cm of the vagina. The central planning target volume (PTV_central_) was created from CTV_central_ with margins of 7, 10 and 10 mm in the left/right, anterior/posterior and superior/inferior direction, respectively. In this study, only pelvic treatment was investigated including elective (CTV_E_) and malignant (CTV_N_) pelvic lymph nodes. The lymph node planning target volume (PTV_E-N_) was created by a 5 mm isotropic expansion of the lymph node CTVs. No additional margin was created for MTV_50_. The bladder, rectum, sigmoid, cauda equina and small bowel (including the entire potential space up to the upper level of the L4 vertebrae) were defined as OARs.

The standard non-SIB plans were normalised to a 1.8 Gy mean dose for the PTV_union_ (union of PTV_central_ and PTV_E-N_) per fraction. For local dose escalation, MTV_50_ was simultaneously boosted to 2.8 Gy per fraction. To avoid de-escalation of the surrounding target volume, PTV_union_ subtracted with a 10 mm isotropic expansion of GTV_tumour_ was used for normalization in the SIB plans. All plans had to fulfil the criteria of PTV_union_ having a D_98_ ≥ 95 % of the prescribed dose. The MTV_50_ D_98_ was also evaluated in the SIB plans. In the optimization, to reduce OAR doses, one dose volume histogram (DVH) constraint was applied to limit maximum dose and additional individually adjusted constraints used to reduce intermediate doses. For each patient, the standard homogeneous plan was created prior to the dose escalated SIB-plan, where the latter was optimized with standard DVH constraints scaled according to 10 fractions. For the relevant OARs, DVH parameters V_30Gy_, V_45Gy_ and D_2cm_^3^ were calculated for all 28 fractions and used together with the D_98_ criteria for PTV_union_ in plan evaluation. Population based DVHs (including standard deviations) were created for comparing the total dose from the short-course SIB to the standard approach for both VMAT and IMPT.

All photon plans were created with the volumetric intensity modulated arc therapy (VMAT) technique Rapid-Arc (Varian Medical Systems, Palo Alto, CA). Each plan consisted of two full arcs with collimator angles of 30° and 330° and one isocenter. A 6 MV photon beam from a Varian Clinac iX with a 120 leaf Millenium MLC (5 mm and 10 mm central and outer resolution, respectively) was used with a 2.5 mm dose calculation grid.

For proton therapy, intensity modulated proton therapy (IMPT) was considered using two opposing lateral fields and one posterior field. In this study, multifield optimization (MFO) was applied, optimizing spots from all three fields simultaneously. Beam line data with a nominal energy range of 70 to 250 MeV were available for treatment planning. The dose distribution was calculated using a 2.5 mm dose grid and assuming a generic relative biological effectiveness (RBE) of 1.1 [15]. Furthermore, a spot spacing of 5 mm was used together with 5 mm circular lateral margins and proximal and distal margins of 5 mm to account for range uncertainties.

The current study is based on pre-treatment PET/CT images and changes in patient anatomy may impact the doses received by the target volumes and OARs. To study the dosimetric impact of tumour shrinkage, OARs (bladder, rectum and sigmoid) were shifted systematically towards the tumour as a single direction expansion of the respective organ volumes in the dose planning system. It was assumed that the tumor is spherical and isotropically shrinking, with a one-to-one association between tumour radius reduction and OAR shift. Based on the mean tumour volume found for patients included in this study and potential tumour regressions of 30 and 50 % [16, 17] OAR shifts of 3 mm and 5 mm, respectively were simulated.

### Statistical analysis

Student’s paired *t*-tests were used for statistical evaluation of DVH parameters in comparing the short-course SIB approach to standard therapy and VMAT to IMPT. In all statistical evaluation, *p*-values below 0.05 were considered statistically significant and *p*-values below 0.001 truncated and displayed as *p* < 0.001.

## Results

**Fig. 2:**
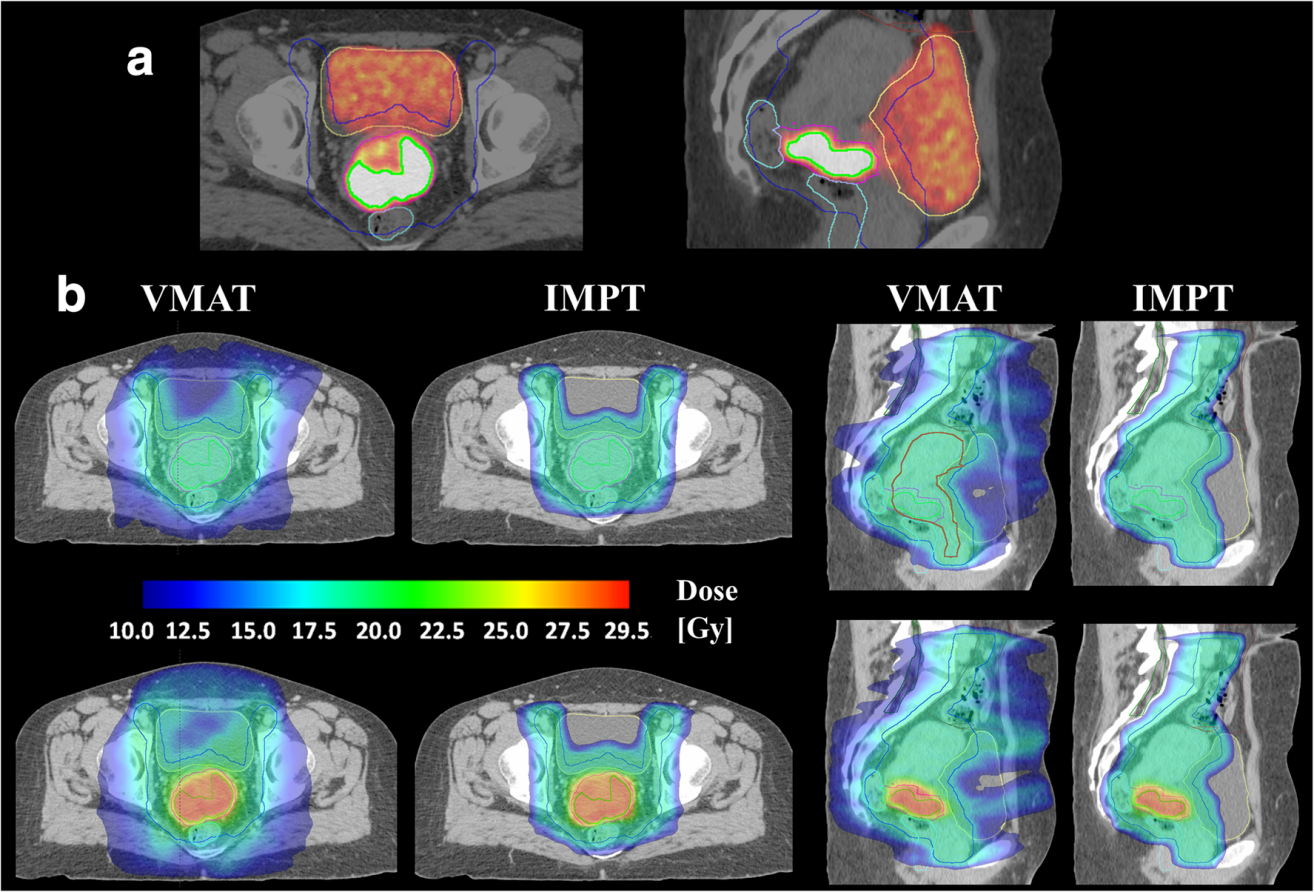
**a** Axial and sagittal views of the FDG PET/CT scan for one patient (GTV_tumour_ volume 66 cm^3^, MTV_50_ volume 31 cm^3^ and SUV_max_ 31). Target volumes PTV_union_ (blue), GTV_tumour_ (*pink*) and MTV_50_ (*green*) and OARs bladder (*yellow*), rectum (*light blue*) and bowel (*brow*n) outlined. **b** Axial and sagittal views of the obtianed dose distributions. Fractions 1–10 are shown for both VMAT and IMPT plans, comparing a standard homogenious plan (*top*) to the corresponding short-course SIB with additional 1.0 Gy per fraction to MTV_50_ (*bottom*)

For the patients included, MTV_50_ had a mean volume of 35 ± 6 cm^3^ as compared to average volumes for the GTV_tumour_ and PTV_union_ of 69 ± 9 cm^3^ and 1474 ± 42 cm^3^, respectively. SUV_max_ ranged from 12 to 31 with a median value of 23. Figure 2 shows axial and sagittal images and dose distributions for one case, comparing 10 SIB fractions to 10 standard non-SIB fractions for both VMAT and IMPT. For this case the dose escalation only had a minor effect on the surrounding volumes for both modalities, also for OARs situated close to the boost volume. In general, the dose escalation was straightforward to implement for both VMAT and IMPT, with a D_98_ ≥ 95 % for MTV_50_ being achieved in all SIB plans. This means that the MTV_50_ was successfully dose escalated from 18 Gy to 28 Gy during the first 10 fractions regardless of modality. Pair wise statistics showed the MTV_50_ D_98_ to be higher in IMPT plans than VMAT plans (*p* < 0.05), but the population based mean values were very similar; 27.3 Gy and 26.9 Gy, respectively.

**Fig. 3:**
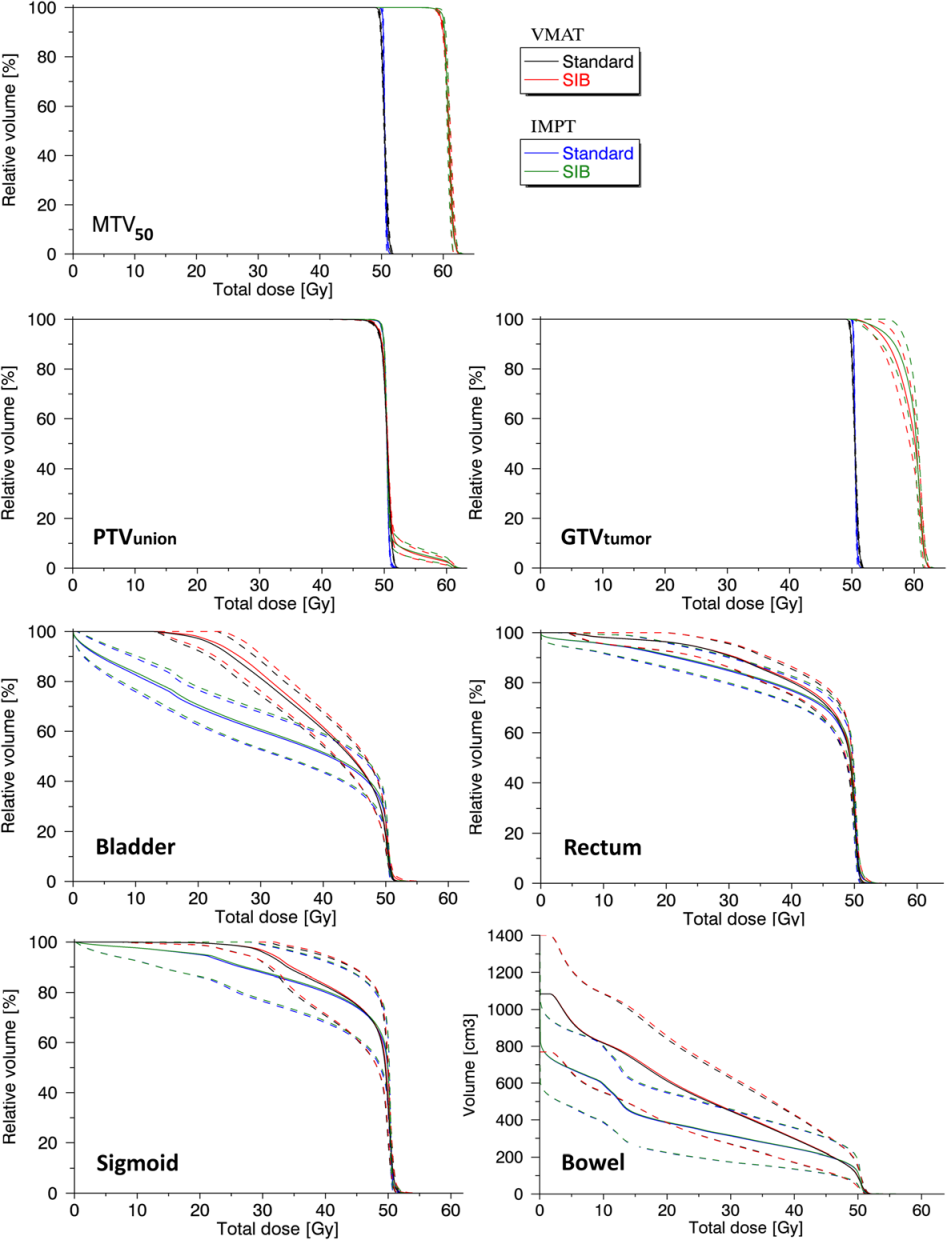
Population based DVHs for all 10 patients for target volumes and OARs (mean in solid lines and mean +/− 1 SD in dotted lines). Standard external beam therapy with 28 fractions of 1.8 Gy to PTV_union_ is compared to the total dose using the short-course approach (SIB) with 10 fractions with 2.8 Gy to MTV_50_ followed by 18 standard fractions for both VMAT and IMPT

Figure 3 shows population based DVHs for target volumes and OARs for both photon and proton therapy. The sum of the short-course SIB (10 fractions) and the subsequent 18 standard fractions is compared to a standard, 28 fraction non-SIB approach. For GTV_tumour_ and PTV_union_, the inter-patient variation is small in the standard approach with adequate coverage for both VMAT and IMPT. In the novel approach inter-patient variation is greater particularly for GTV_tumour_, as the relative size of the boost volume varies between patients. Target volume coverage, however, remains good when dose escalating. Looking at the OARs, all the dose distributions are almost identical when comparing the SIB approach to the standard approach. For bladder however, a slight difference is visible in the low to intermediate dose-range with about 1 Gy increase for the SIB as compared to standard fractionation for VMAT. In general however, patient to patient variations in OAR doses due to anatomical differences were up to about 10 Gy (Fig. 3), which are far greater than the increase from dose escalation. A summary of OAR DVH parameters V_30Gy_, V_45Gy_ and D_2cm_^3^ is presented in Table 1, where the total treatment following the short-course approach is compared to standard fractionation for both modalities. Pair wise statistics showed significantly higher values for many parameters, but with a mean difference less than 2 % in all cases except for V_30Gy_ in bladder using VMAT (3 % increase).

**Table 1:** Overall dose volume histogram parameters for OARs

		Bladder			Rectum			Sigmoid			Bowel^*^		
Plan/Param		V_30Gy_ [%]	V_45Gy_ [%]	D_2cc_ [Gy]	V_30Gy_ [%]	V_45Gy_ [%]	D_2cc_ [Gy]	V_30Gy_ [%]	V_45Gy_ [%]	D_2cc_ [Gy]	V_30Gy_ [cm^3^]	V_45Gy_ [cm^3^]	D_2cc_ [Gy]
VMAT	Stand.	81.3 ± 2.2	48.0 ± 2.2	51.2 ± 0.1	90.8 ± 1.5	72.1 ± 1.9	50.9 ± 0.1	96.0 ± 1.3	75.0 ± 4.4	51.0 ± 0.1	448 ± 56	218 ± 31	51.7 ± 0.1
	SIB	83.5 ± 2.3^†^	48.7 ± 2.3	51.9 ± 0.3^†^	91.1 ± 1.5	73.1 ± 2.0^†^	51.4 ± 0.2^†^	96.6 ± 1.4	75.3 ± 4.4	51.1 ± 0.1	452 ± 59	220 ± 31	51.8 ± 0.2
IMPT	Stand.	60.1 ± 2.4	44.3 ± 2.3	51.0 ± 0.1	84.7 ± 1.7	70.3 ± 1.7	50.9 ± 0.1	87.7 ± 3.6	74.1 ± 4.0	50.8 ± 0.1	313 ± 44	206 ± 30	51.3 ± 0.1
	SIB	60.8 ± 2.4^†^	45.1 ± 2.3^†^	51.2 ± 0.2^†^	85.2 ± 1.7^†^	71.0 ± 1.8^†^	51.2 ± 0.2^†^	88.2 ± 3.5	74.4 ± 4.0	50.9 ± 0.1	316 ± 45^†^	208 ± 31^†^	51.6 ± 0.3

Dose volume histogram parameters comparing the overall treatment following the short-course concept to a standard fractionated schedule

Values are mean ± standard deviation of the mean

*Abbreviations*: *Stand* standard plan of 1.8Gy X 28, *SIB* sum of 10 fraction SIB and 18 fraction standard plan

^*^Entire potential space within irradiated area,

^†^Significant pair wise difference between Stand and SIB parameter

In the dosimetric sensitivity analysis, OAR shifts of 3 mm and 5 mm towards the primary tumour were simulated. The resulting D_2cm_^3^ per fraction (EQD2) in the SIB plans for the shifted OARS were compared to corresponding values for the planned static situation (Table 2). For bladder and rectum, such shifts gave a D_2cm_^3^ increase per fraction from around 0.2 Gy to 0.4 Gy compared to the planned, original SIB.

**Table 2:** Dosimetric sensitivity of OAR clinical maximum dose, D_2cm_^3^

	Bladder [Gy]		Rectum [Gy]		Sigmoid [Gy]	
Param/Plan	VMAT	IMPT	VMAT	IMPT	VMAT	IMPT
Standard	1.77 ± 0.00	1.75 ± 0.00	1.75 ± 0.00	1.75 ± 0.01	1.75 ± 0.01	1.74 ± 0.00
SIB	1.92 ± 0.04	1.80 ± 0.01	1.85 ± 0.02	1.82 ± 0.02	1.81 ± 0.02	1.78 ± 0.01
SIB 3 mm shift	2.11 ± 0.08	2.02 ± 0.06	2.04 ± 0.04	2.00 ± 0.05	1.93 ± 0.05	1.88 ± 0.05
SIB 5 mm shift	2.23 ± 0.10	2.20 ± 0.09	2.16 ± 0.06	2.15 ± 0.06	2.02 ± 0.08	1.97 ± 0.08

Dosimetric sensitivity analysis estimating increase of D_2cm_^3^ to OARs from shifts towards the high dose area potentially caused by tumour shrinkage

Values are EQD2 per fraction (α/β = 3) and in mean ± standard deviation of the mean

## Discussion

In this study a PET-based short-course approach for dose escalation in treatment of locally advanced cervical cancer has been presented. The results show that both photon and proton therapy allows for a 10 Gy dose escalation of the boost-volume during the 10 first fractions with only a minor increase of OAR doses. Comparing VMAT to IMPT for standard homogeneous plans, our results were in line with previous planning studies for cervical cancer with reduced low to intermediate OAR doses using protons [18–20]. In dose escalation however, IMPT did not show an additional advantage over VMAT. This follows as both modalities succeeded in increasing the dose to the FDG avid tumour sub-volume without noteworthy increasing OAR doses compared to a standard homogeneous plan.

Although cervix tumours shrink during treatment [16, 17], a significant number of patients still present with large or asymmetric tumours at time of brachytherapy. This remains a challenge as these patients are expected to benefit from higher target doses [21]. At the same time, it is more difficult to achieve full target coverage for large tumours. Increased tumour remission before brachytherapy is therefore desired, particularly as many centers worldwide do not offer the possibility of interstitial implants. Dose escalation delivered 2–3 weeks prior to brachytherapy may provide such additional tumour shrinkage, being an important incentive for the short-course approach. Dose escalation of an FDG avid sub-volume was investigated in this study, but other boost volume definitions may also be considered. With a marginal increase in OAR D_2cm_^3^ from the EBRT dose escalation, only small changes to standard dose constraints are expected for brachytherapy. The suggested concept for external beam dose escalation may therefore be feasible, potentially facilitating for improved brachytherapy.

Anatomical changes occur during treatment of cervical cancer and are of particular concern when applying a SIB with high fraction doses and steep dose gradients. Even though the cervix shows less motion than the uterus [22], daily image guidance with soft tissue contrast will be vital for clinical implementation of any dose escalation. Cone beam CT imaging (CBCT) with subsequent couch correction may be used to avoid overlap between the high dose area and OARs. It can nevertheless be treatment sessions where adequate couch corrections are not possible and in these cases the standard plan can serve as a robust backup plan similar to the plan of the day concept [23]. A drinking protocol and markers for targeting of the boost volume [23] may also be necessary to reduce geometric uncertainties. Intra fraction motion is generally found to be smaller than inter fraction motion [22] with cervical displacement found to be less than 5 mm in most cases [24]. This motion can however currently not be corrected for and may also increase slightly with treatment time [22], thus potentially being larger during IMPT than VMAT.

The dosimetric effects of anatomical changes will be greater for IMPT than VMAT as proton stopping power is more sensitive to tissue changes than the corresponding photon interaction coefficients. These effects have previously been investigated for standard and dose escalated intensity modulated radiotherapy (IMRT) of cervix cancer [25, 26] and for IMPT with a SIB for prostate cancer [27]. Herrera et al. [26] analyzed dose escalation of a central target volume including the uterus over 28 fractions using deformable registration of weekly CBCT images. They found, compared to planned values, overdosage of adjacent OARs correlating with tumour shrinkage and recommended frequent plan adaptation. In our approach however, a smaller PET based volume confined well within the GTV is to be dose escalated. A greater distance between the high dose area and OARs gives lower OAR doses, and our strategy is thus expected to be less sensitive to random organ motion. Also, dose escalation is limited to the 10 first fractions, leaving less time for systematical anatomical changes. As the tumour may regress during the course of the treatment, adjacent OARs may experience a systematic shift towards the high dose area in addition to random day to day variations. However, as tumour shrinkage is expected to be limited in the two first weeks of treatment [16, 17, 26], the extra OAR dose estimated from this effect (Table 2) may only potentially be achieved for a few fractions towards the end of dose escalation. After completion of dose escalation however, repeated imaging should be considered for possible adaptation of the following standard non-SIB plan. Such timing would also be consistent with previous clinical trials in adaptive PET based dose painting of head and neck cancer where plan adaptation were implemented after 10 treatment fractions [28, 29].

In dose painting, biological imaging reflecting the spatial distribution of radiobiological properties is used to prescribe an inhomogeneous dose distribution to the tumour [30]. It may be carried out as dose painting by numbers (DPBN) [31], where the voxel dose is determined by the corresponding biologic voxel value, or as dose painting by contours (DPBC), where an image based biologic target volume is dose escalated. The technique has proven feasible and there are several ongoing and completed phase 1 and 2 studies applying FDG PET based dose-painting for head and neck and non-small-cell lung cancer (NSCLC) [32]. The two-level DPBC approach presented here is to our knowledge the first investigation of PET-based dose painting for cervical cancer. The choice of 50 % of SUV_max_ as a threshold in boost volume segmentation is consistent with previous clinical implementation of DPBC for NSCLC and head and neck cancer [29, 33]. Further investigations of the short-course concept for PET based dose escalation may also include DPBN as it, compared to DPBC, is suggested to be less sensitive to PET reconstruction parameters [34].

The usage of a SIB during EBRT of locally advanced cervical cancer is not uncommon, but the aim has typically been to treat gross disease in the parametria or metastatic lymph nodes [35, 36]. The motivation is to reduce total treatment time [37] compared to the conventional approach with sequential boosting of lymph nodes. Acceptable acute toxicity and promising tumour control is reported [35, 36], demonstrating that SIB is a feasible approach in treatment of cervical cancer. There are other studies investigating high external fraction doses to the primary tumour using photons and protons, but then instead of and not in addition to brachytherapy [38, 39]. A recent analysis reviewing treatment outcome for different boost modalities, did however find a significantly increased mortality risk in patients receiving external boost only [40]. The dose escalation concept suggested here is therefore not meant to replace brachytherapy. It is rather meant to facilitate for improved brachytherapy by possibly increasing tumour shrinkage.

## Conclusions

A short-course approach to PET based dose escalation has been presented and found feasible. Clinical implementation of this concept will require the use of currently available image guidance techniques. In addition to expected increase of tumour control, this approach may enhance tumour shrinkage before brachytherapy. Thus, this novel treatment concept may prove clinically valuable, in particular for patients with large or asymmetric tumours.

## Abbreviations

BT: brachytherapy
CBCT: cone beam CT imaging
CT: computed tomography
CTV: clinical target volume
DPBC: dose painting by contours
DPBN: dose painting by numbers
DVH: dose volume histogram
EBRT: external beam radiotherapy
EQD2: 2 Gy equivalent dose
FDG: 18F-fluorodeoxyglucose
GTV: gross target volume
HDR: high dose rate
HR-CTV: high risk CTV
IGABT: image guided adaptive brachytherapy
IMPT: intensity modulated proton therapy
IMRT: intensity modulated radiotherapy
LACC: locally advanced cervical cancer
MFO: multifield optimization
MR: magnetic resonance
MTV: metabolic target volume
NSCLC: non-small-cell lung cancer
OAR: organ at risk
OSEM: ordered subset expectation maximization
PET: positron emission tomography
PTV: planning target volume
SIB: simultaneous integrated boost
SUV: standardised uptake value
TPS: treatment planning system
VMAT: volumetric intensity modulated arc therapy
